# Navigating Diagnostic Complexities and Treatment Strategies of Moyamoya Syndrome: A Case Report

**DOI:** 10.7759/cureus.73242

**Published:** 2024-11-07

**Authors:** Li Li Kwan, Anna Misyail Abdul Rashid, Mohamad Syafeeq Faeez Md Noh, Fan Kee Hoo, Liyana Najwa Inche Mat

**Affiliations:** 1 Department of Internal Medicine, Faculty of Medicine and Health Sciences, Universiti Putra Malaysia, Serdang, MYS; 2 Department of Neurology, Faculty of Medicine and Health Sciences, Universiti Putra Malaysia, Serdang, MYS; 3 Department of Radiology, Faculty of Medicine and Health Sciences, Universiti Putra Malaysia, Serdang, MYS

**Keywords:** atherosclerosis, cerebral bypass, collaterals, moyamoya syndrome, stroke

## Abstract

Moyamoya syndrome (MMS) is characterized by progressive narrowing of intracranial arteries and the development of collateral vessel networks, often presenting with recurrent ischemic or hemorrhagic strokes. MMS poses significant challenges in diagnosis due to its overlapping symptoms with other cerebrovascular conditions. Treatment aims to improve cerebral blood flow, reduce symptom frequency, and prevent future strokes. Management requires a multidisciplinary approach, including medical optimization and potential surgical intervention. This case report discusses a 37-year-old woman with poorly controlled type 2 diabetes and hyperlipidemia who experienced multiple strokes before being diagnosed with MMS. Initial symptoms included intermittent headaches, hemiparesis, and slurred speech. The presence of underlying metabolic conditions led to an initial assumption of atherosclerosis as the primary cause of her strokes. This case underscores the diagnostic challenge of distinguishing MMS from more common vascular conditions, highlighting the need for careful evaluation when typical risk factors like atherosclerosis do not fully explain recurrent cerebrovascular events.

## Introduction

The term "moyamoya" is derived from a Japanese word meaning "puffy, obscure, or hazy like a puff of smoke in the air," describing the characteristic appearance of collateral vessels on angiography. Moyamoya disease (MMD), also known as primary or idiopathic MMD, refers to patients who exhibit moyamoya angiographic findings with no associated conditions, though they may have genetic susceptibilities. In contrast, moyamoya syndrome (MMS) refers to patients with moyamoya angiographic findings who also have an associated medical condition, such as diseases affecting the arteries around the circle of Willis (e.g., meningitis, brain tumor, trauma, metabolic diseases, hematological conditions, vasculitis, and autoimmune diseases) [1].

The pathophysiology of moyamoya involves vessel wall thickening and angiogenesis, leading to arterial stenosis and the development of small vessel collateralization [2]. MMS commonly presents with recurrent ischemic strokes, transient ischemic attacks, and hemorrhagic strokes, along with less frequent symptoms like seizures and headaches. In children, ischemic strokes predominate due to arterial narrowing, while in adults, hemorrhagic strokes are more common, resulting from the rupture of fragile collateral vessels [3,4]. The gold standard for diagnosis is cerebral angiography, which shows stenosis or occlusion in the terminal portion of the intracranial internal carotid artery (ICA) and the formation of abnormal vascular networks or collaterals [5].

The primary goal of treatment is to improve cerebral blood flow (CBF), reduce symptom frequency, and prevent future strokes by promoting collateral circulation. Achieving long-term functional improvement is crucial. Critical factors in evaluating treatment success include CBF and cerebral perfusion pressure (CPP), which help determine how well interventions prevent ischemic events and maintain adequate brain oxygenation. These measures guide the effectiveness of therapies in optimizing blood supply and minimizing stroke risk over time [6,7].

## Case presentation

**Figure 1:**
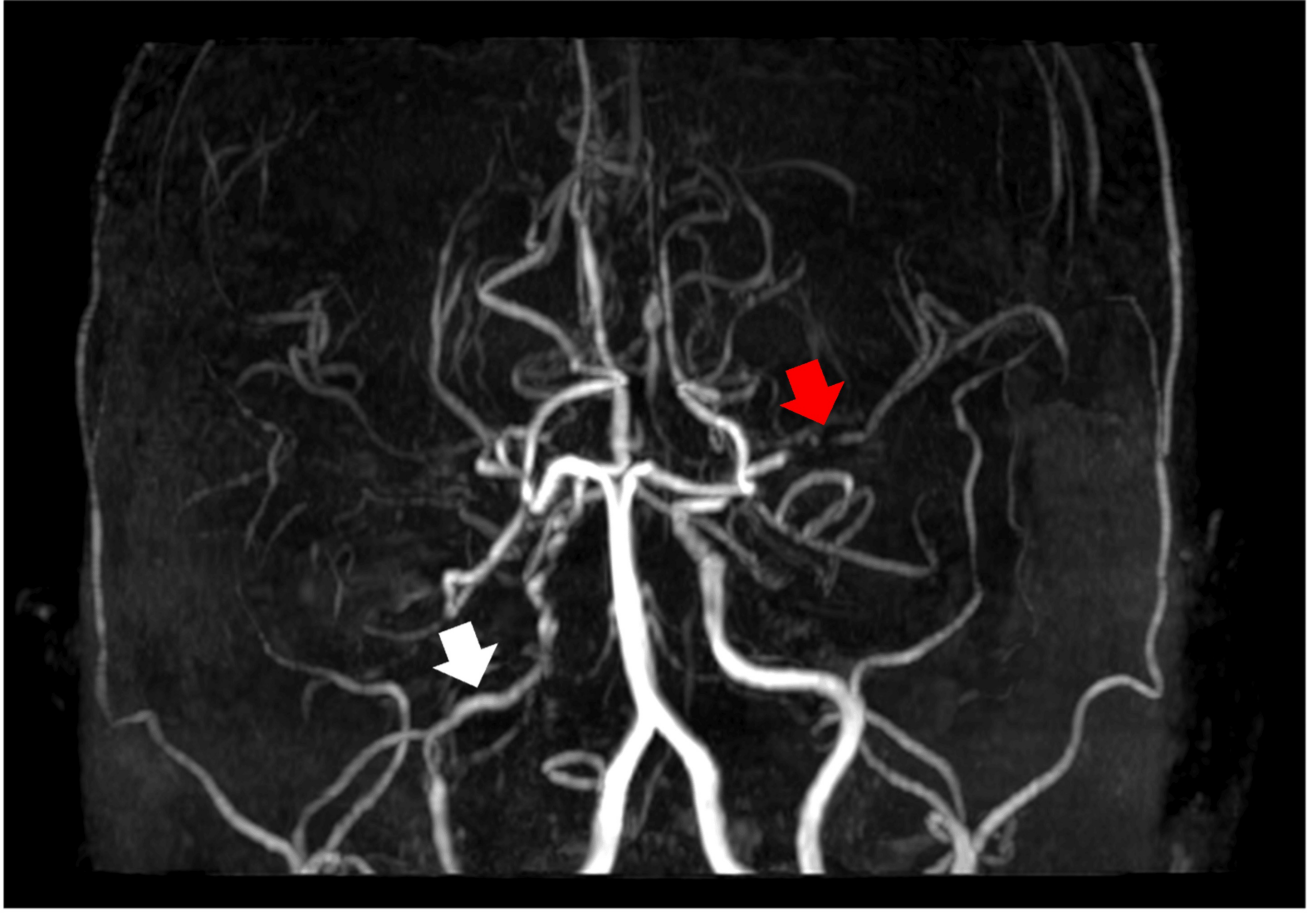
MRA showing diffuse stenosis of the right intracranial ICA (white arrow) and occlusion of the proximal segment of the right MCA with faint signal at the distal territories (red arrow). ICA: Internal carotid artery; MRA: Magnetic resonance angiogram; MCA: Middle cerebral artery

A 37-year-old woman with a history of poorly controlled type 2 diabetes, complicated by microvascular and macrovascular target organ damage and hyperlipidemia, initially presented with a one-week history of headaches, right hemiparesis, and slurred speech. These symptoms were intermittent, occurring and resolving over several episodes throughout the year, with increasing frequency. On examination, there was no motor deficit with normal tone and reflexes. Sensation was intact, with no cerebellar signs and normal gait. The National Institutes of Health Stroke Scale (NIHSS) score was zero. The brain's MRI showed old infarcts at the right frontal lobe and left caudate nucleus. Magnetic resonance angiogram (MRA) showed stenosis of the right intracranial ICA with stenosis of the left middle cerebral artery (MCA) and non-visualization of the rest of the left anterior cerebral arteries (ACA) segments (Figure 1).

**Figure 2:**
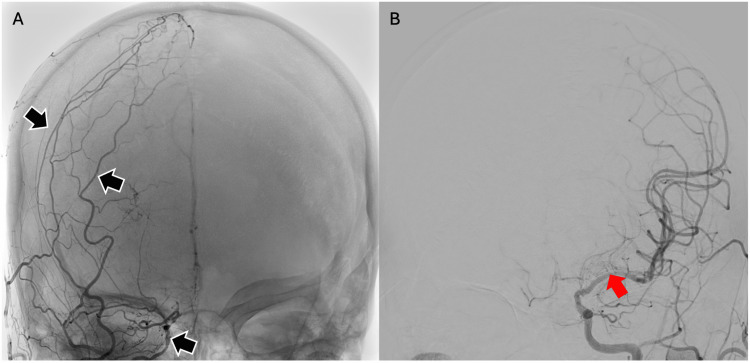
Cerebral angiogram showing: (A) Small right intracranial ICA with evidence of external carotid artery-internal carotid artery (ECA-ICA) collaterals (black arrows). (B) Short segment stenosis involving the left MCA is noted (red arrow). ICA: Internal carotid artery; MCA: Middle cerebral artery

Following this, a cerebral angiography was performed, showing a small right ICA with multiple external carotid artery-internal carotid artery (ECA-ICA) collaterals. The left ICA and ECA were also reported as normal, but there was atherosclerotic disease in the left MCA (Figure 2). Metabolic workup indicated hyperlipidemia and suboptimal blood sugar control (total cholesterol: 5.9 mmol/L, triglycerides: 2.9 mmol/L, low-density lipoprotein (LDL): 3.28 mmol/L, and hemoglobin A1c (HbA1c): 12.4%), liver function test and renal profile were normal (Table 1). As her NIHSS was zero, we decided on the best medical therapy. She was treated with aspirin and statin.

**Table 1:** Investigations sent for the patient Anti-dsDNA: Anti-double stranded DNA; P-ANCA: Perinuclear anti-neutrophil cytoplasmic antibodies; C-ANCA: Cytoplasmic antineutrophil cytoplasmic antibody; TSH: Thyroid stimulating hormone

Laboratory parameters	Patient range	Normal range
Total cholesterol	5.9 mmol/L	0-5.2 mmol/L
Triglycerides	2.9 mmol/L	0.08-1.7 mmol/L
Low-density lipoprotein (LDL)	3.28 mmol/L	0 mmol/L
HbA1c	12.4%	<6.3%
Hemoglobin	12 g/dL	13.0-17.0 g/dL
White cell counts	5.3 x 10^3^/uL	4.00-10.00 x 10^3^/uL
Platelets	390 x 10^3^/uL	150-410 x 10^3^/uL
Albumin	38 g/L	34-48 g/L
Alanine transaminase (ALT)	15 U/L	0-55 U/L
Alkaline phosphatase (ALP)	107 U/L	40-150 U/L
Total bilirubin	15 umol/L	3.4-20.5 umol/L
Urea	3 mmol/L	3.5-7.2 mmol/L
Creatinine	70 umol/L	50.4-98.1 umol/L
Sodium	139 mmol/L	136-145 mmol/L
Potassium	4.9 mmol/L	3.5-5.1 mmol/L
Antinuclear antibody (ANA)	Negative	Negative
Complement level C3	1.55	0.83-1.93 g/L
Complement level C4	0.34	0.15-0.57 g/L
Anti-dsDNA	<0.6 IU/mL	<10 IU/mL
P-ANCA	Negative	Negative
C-ANCA	Negative	Negative
Rheumatoid factor (RF)	Negative	Negative
Lupus anticoagulant	27.1 seconds	24.9-33.0 seconds
Anticardiolipin Antibody	<2.0 IU/mL	<12 IU/mL
Beta 2 microglobulin	<20 U	<21 U
Anti-thrombin III	118.0%	83.00-128.00%
Protein C	148.8%	70.0-140.0%
Protein S	63.9%	54.7-123.7%
Homocysteine	12 umol/L	<15 umol/L
T4	15 pmol/L	10-26 pmol/L
TSH	0.89 mIU/L	0.4-4.0 mIU/L

**Figure 3:**
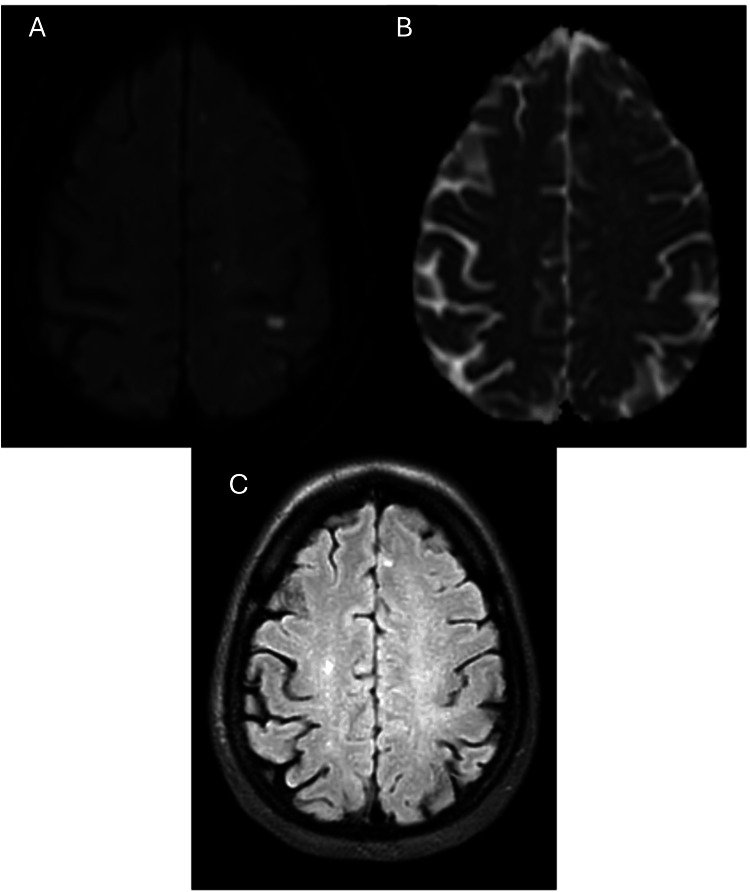
A repeat MRI of the brain revealed: (A) Axial DWI image (b=1000), with corresponding low signal on the axial ADC image (B) demonstrating acute infarct in the left high parietal and centrum semiovale regions. (C) The corresponding axial FLAIR image shows an increased signal at the same region. DWI: Diffusion-weighted imaging; FLAIR: Fluid-attenuated inversion recovery; ADC: Apparent diffusion coefficient

She experienced her first clinical stroke nine months later, presenting with right hemiparesis involving the right upper and lower limbs with slurred speech. Examination showed a power of 4/5 in the right upper and lower limbs, and the NIHSS scored 2/42 (mild sensory loss and drift over the right lower limb). A repeat MRI of the brain revealed an acute infarct in the left high parietal and centrum semiovale regions (Figure 3), with essentially unchanged MRA findings as the previous MRA showing right ICA, left MCA, and left ACA intracranial atherosclerotic disease (ICAD) (Figure 1). Dual antiplatelet therapy with Cardiprin and clopidogrel was initiated, and her medications were optimized to minimize stroke risk. She was discharged with an NIHSS score of 1/42 (drift over the right lower limb) and an outpatient appointment due to the mild nature of the stroke. Clopidogrel was discontinued after one month, and Cardiprin was continued.

**Figure 4:**
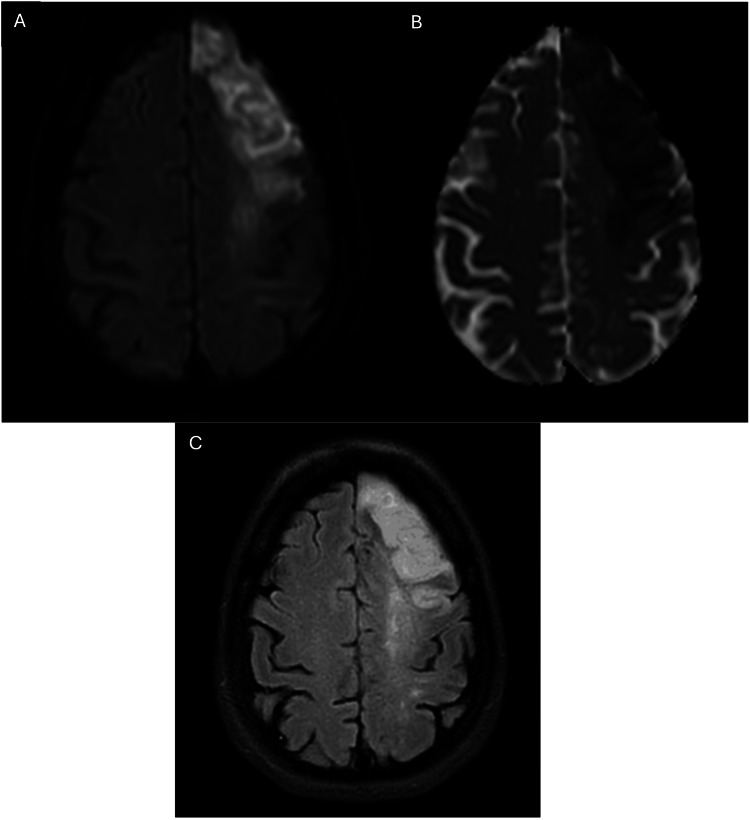
MRI of the brain showing: (A) Axial DWI image (b=1000), with corresponding low signal on the axial ADC image (B) demonstrating a left anterior cerebral artery (ACA) territory infarct. (C) The corresponding axial FLAIR image shows an increased signal at the same region. DWI: Diffusion-weighted imaging; FLAIR: Fluid-attenuated inversion recovery; ADC: Apparent diffusion coefficient

However, she suffered a second stroke nine months later, presenting with right-sided hemiparesis despite compliance with medication. On examination, her blood pressure was 188/109 mmHg, heart rate 89 beats per minute, and blood glucose 18 mmol/L. Neurological examination showed a motor deficit in the right upper and lower limbs with NIHSS score of 4/42 (right upper and lower limbs drift - 1 point each, facial asymmetry - 1 point, and mild aphasia - 1 point). MRI of the brain showed an acute left frontal infarction with a diffusion-weighted imaging-fluid-attenuated inversion recovery (DWI-FLAIR)-matched lesion (Figure 4). She was hospitalized and reinitiated on a double antiplatelet regime.

Secondary workup, including autoimmune screening (antinuclear antibody, C3C4, antineutrophil cytoplasmic antibody, rheumatoid factor, lupus anticoagulant, thrombophilia screening), homocysteine, and thyroid function tests were normal (Table 1), as were echocardiography (ECHO), ECHO bubble test, transcranial doppler ultrasound, and Holter. Cholesterol levels and diabetic control were optimal after medication optimization during this admission.

**Figure 5:**
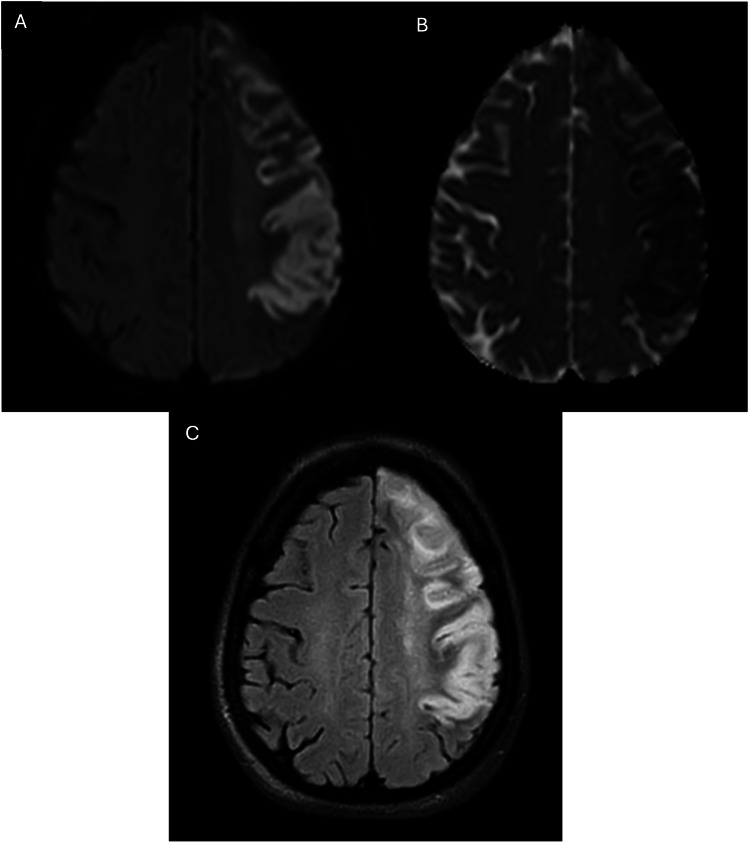
MRI of the brain showing: (A) Axial DWI image (b=1000), with its corresponding axial ADC image. (B) exhibiting expansion of the infarct to involve the left middle cerebral artery (MCA) region, affecting the left parietal lobe. Some evidence of pseudonormalization is already seen. (C) The corresponding axial FLAIR image shows an increased signal at the same region. DWI: Diffusion-weighted imaging; FLAIR: Fluid-attenuated inversion recovery; ADC: Apparent diffusion coefficient

**Figure 6:**
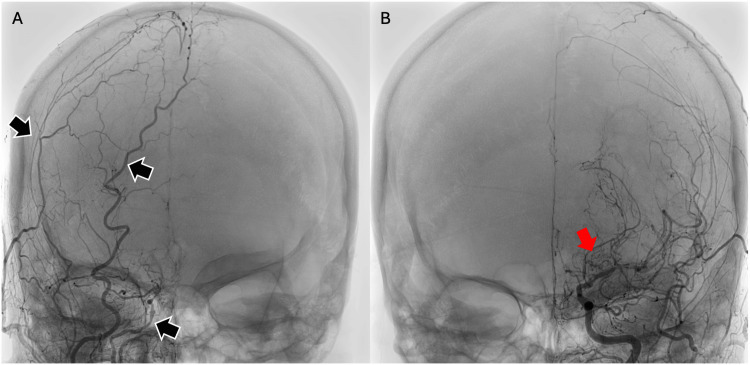
A repeat cerebral angiogram showing: (A) small right intracranial ICA with multiple ECA-ICA collaterals (black arrows); (B) severe stenosis at the mid to distal left MCA with extensive small collateral arterial networks arising from the lenticulostriate and choroidal arteries, resembling a "puff of smoke" appearance (red arrow). ECA-ICA: External carotid artery-Internal carotid artery

Unfortunately, she deteriorated in the ward one week later despite optimal medication. She developed aphasia and dense hemiparesis in the right upper and lower limbs, with power reduced to 0/5 and 3/5, respectively, increasing her NIHSS score to 14. A repeat brain MRI showed an acute infarction expanding to the left frontoparietal (MCA) territory (Figure 5). A repeat cerebral angiogram showed a small right intracranial ICA with multiple ECA-ICA collaterals and severe stenosis at the mid to distal left MCA with multiple ECA-ICA collaterals and extensive small collateral arterial networks arising from the lenticulostriate and choroidal arteries, resembling a "puff of smoke" appearance (Figure 6). Multilevel bilateral anterior circulation stenoses led to a diagnosis of MMS as a cause of her clinical deterioration.

After a multidisciplinary team meeting among the neurologists, neurosurgeons, and interventional radiologists, the etiology of the infarct was attributed to MMS. The main concern was the rapid progression of neurological deficit and evidence of expanding infarction despite optimization of medication and risk factors, which included a trial of ticagrelor. Hence, the neurosurgeon suggested bypass surgery to augment the flow to the infarcted area, thus facilitating cerebrovascular flow recovery. A left craniotomy, superficial temporal artery to middle cerebral artery (STA-MCA) bypass, and encephaloduroarteriosynangiosis (EDAS) were carried out without complication. The patient showed positive outcomes after surgery, with motor function in the right lower limb improving to grade 4/5, with an NIHSS score of 9 on day five post-operatively. She underwent intensive rehabilitation, and her general condition continued to recover. At three months post-operatively, she was able to perform activities of daily living with minimal assistance and improvement of NIHSS to 6.

## Discussion

MMS presents significant challenges in diagnosis and management due to its complex and multifactorial nature. It is characterized by the progressive narrowing of large intracranial arteries and the secondary development of prominent small-vessel collaterals. The Research Committee on Moyamoya Disease (RCMD) 2021 has updated the diagnostic criteria of MMD and MMS, stating that both bilateral and unilateral vessel diseases can be diagnosed as MMS. However, it is necessary to exclude atherosclerotic diseases [5]. This may pose a challenge, particularly in unilateral disease and elderly populations. Thus, the pattern of vessel wall enhancement on MRA may provide valuable supportive information.

In MMS, the narrowing of the lumen is due to intimal thickening and, at the same time, a reduction in their outer diameter on heavy T2-weighted MRI; on the other hand, in the case of MCA stenosis caused by atherosclerosis, the lumen is only narrowed, and the outer diameter of the vessel does not shrink [5,7,8]. Although these findings are helpful, the shrinking of the artery does not occur until a later stage of disease, in which case the cerebral angiogram is often recommended. Vessel wall imaging of MMS generally reveals negative remodeling with reduced diameter and heterogeneous enhancement, distinguishing it from MMD, where compensatory vessel enlargement (positive remodeling) is absent [8-10].

This case highlights the difficulties encountered in recognizing and treating the condition, especially when it coexists with other systemic diseases like diabetes and hyperlipidemia. This is in line with current literature suggesting that MMS is often underdiagnosed or misdiagnosed in patients with stroke [11]. In our patient, intermittent symptoms of headache, hemiparesis, and slurred speech, coupled with her underlying diabetes and hyperlipidemia, initially suggested atherosclerotic cerebrovascular disease rather than MMS. The initial MRI scans revealed severe luminal narrowing and old infarcts, which could be misinterpreted as typical atherosclerotic changes. However, the progressive nature of the stenosis and the development of extensive collateral networks were key indicators of MMS, highlighting the importance of a diagnostic cerebral angiogram [12].

The management of MMS can be divided into non-invasive and invasive. Non-invasive treatments include the use of antiplatelets (i.e., aspirin and clopidogrel), vasodilators (i.e., cilostazole), and management of vascular risk factors [6,11]. It is important to note that diabetes is an independent predictor of recurrent ischemic stroke in nonsurgical and surgically treated patients [13]. Despite optimal medical treatment, the disease progression rate is approximately 20% over six years, and the female gender was an independent risk factor for disease progression [14].

As such, the patient's initial management was apt, involving strict risk factor modifications and an antiplatelet regime. However, despite compliance and optimal control, she continued to develop recurrent symptoms, prompting the necessity of invasive treatment such as revascularization surgery. Among the general indications for revascularization are recurrent clinical symptoms due to apparent cerebral ischemia or decreased regional CBF [13,15].

Revascularization techniques include direct procedures like STA-MCA bypass, where an external carotid branch is connected to a cortical artery, and indirect techniques like synangiosis, which promotes collateral circulation using connective tissues [14,15]. However, systematic reviews suggest that STA-MCA bypass improves neurological outcomes with high long-term graft patency and low complication rates, supporting its use in patients under 60 years old who do not respond to medical treatment [16].

Unfortunately, there is a great discrepancy in the literature on the ideal timing of the surgery, where some argue on the merits of early vs delayed revascularization [17,18]. In most cases, early in the disease state, there are usually temporary or mild neurological symptoms, which makes surgery an unappealing choice [18]. Patients usually choose to defer surgery, opting for medical treatment. However, due to natural disease progression, they are bound to develop further neurological deterioration. Furthermore, MMS is a form of non-atherosclerotic vasculopathy, hence the use of antiplatelet or its combination is ineffective to avoid recurrent cerebral infarction, as in our patient [19].

At this point, deciding whether further revascularization therapy is indicated or not is challenging. The literature presents varying opinions on the optimal timing of surgical intervention. Some experts advocate for early revascularization to prevent further ischemic events, while others suggest delaying surgery to reduce the risk of postoperative complications [19,20]. The main conundrum in our patient was further deterioration in clinical symptoms with worsening MRI, prompting urgent treatment. Ultimately, urgent revascularization was deemed necessary, and she responded well to the procedure. The limitation of this report is its focus on a single patient at a single center, underscoring the need for larger, multicenter studies to validate the findings and improve generalizability.

## Conclusions

This case highlights the complexities in diagnosing and managing MMS, particularly when it coexists with other systemic conditions. Increased awareness and early detection are critical, especially in patients with atypical presentations. Advanced imaging techniques and a comprehensive differential diagnosis are essential for accurate evaluation. The primary treatment goals are to restore motor function, improve daily activities, and reduce the overall burden on patients. Early revascularization of the ischemic brain is crucial and can be achieved through medical and surgical interventions. Early rehabilitation also plays a vital role in recovery. A well-structured management plan, including appropriate surgical interventions, is key to enhancing patient outcomes. Ongoing research and advancements in diagnostic and therapeutic strategies are vital for overcoming the challenges associated with this rare cerebrovascular disorder.
